# Neural mechanisms of musical structure and tonality, and the effect of musicianship

**DOI:** 10.3389/fpsyg.2023.1092051

**Published:** 2023-02-09

**Authors:** Lei Jiang, Ruiqing Zhang, Lily Tao, Yuxin Zhang, Yongdi Zhou, Qing Cai

**Affiliations:** ^1^Key Laboratory of Brain Functional Genomics (MOE & STCSM), Affiliated Mental Health Center, School of Psychology and Cognitive Science, East China Normal University, Shanghai, China; ^2^School of Music, East China Normal University, Shanghai, China; ^3^Shanghai High School International Division, Shanghai, China; ^4^School of Psychology, Shenzhen University, Shenzhen, China; ^5^Krieger Mind/Brain Institute, Johns Hopkins University, Baltimore, MD, United States; ^6^Shanghai Changning Mental Health Center, Shanghai, China; ^7^NYU-ECNU Institute of Brain and Cognitive Science, New York University Shanghai, Shanghai, China

**Keywords:** music, tonality, syntax, hierarchical structure, fMRI, informational connectivity

## Abstract

**Introduction:**

The neural basis for the processing of musical syntax has previously been examined almost exclusively in classical tonal music, which is characterized by a strictly organized hierarchical structure. Musical syntax may differ in different music genres caused by tonality varieties.

**Methods:**

The present study investigated the neural mechanisms for processing musical syntax across genres varying in tonality – classical, impressionist, and atonal music – and, in addition, examined how musicianship modulates such processing.

**Results:**

Results showed that, first, the dorsal stream, including the bilateral inferior frontal gyrus and superior temporal gyrus, plays a key role in the perception of tonality. Second, right frontotemporal regions were crucial in allowing musicians to outperform non-musicians in musical syntactic processing; musicians also benefit from a cortical-subcortical network including pallidum and cerebellum, suggesting more auditory-motor interaction in musicians than in non-musicians. Third, left pars triangularis carries out online computations independently of tonality and musicianship, whereas right pars triangularis is sensitive to tonality and partly dependent on musicianship. Finally, unlike tonal music, the processing of atonal music could not be differentiated from that of scrambled notes, both behaviorally and neurally, even among musicians.

**Discussion:**

The present study highlights the importance of studying varying music genres and experience levels and provides a better understanding of musical syntax and tonality processing and how such processing is modulated by music experience.

## 1. Introduction

Throughout the history of humanity, music has been a key component in social and cultural interactions. How people communicate with music, namely how listeners process music syntax has been the subject of investigation in neuroscience. Some studies have suggested parallels between music processing and language processing. Patel (2003) claimed that the comparability of syntactic structure exists in both music and language: as within language, a multilayered organization principle (tones to chords and then to chords progression) governs the listeners to form an abstract-level musical structural understanding (i.e., musical syntax), which does not necessarily require explicit knowledge of music theory. Currently, however, the neural mechanisms of tonal music perception are still uncertain. Some evidence has been provided by studies on Western classical music. The organization of pitches or chords in a classical harmonic musical sequence tends to begin with the main tone or chord, and usually returns to the main tonic or tonic chord at the end. Other genres of music involve different structures, which in turn may entail different processing mechanisms to classical music.

Animal studies have shown that, in marmosets, harmonic template neurons sensitive to the spectral regularity of harmonic complex sounds are distributed across the primary auditory cortex and the neighboring primary-like rostral area (Feng and Wang, 2017). In humans, widely distributed frontal and temporal regions have been involved in the processing of classical music. Among these regions, the left inferior frontal gyrus (IFG) has been suggested to be the most important site offering computational resources for both linguistic and musical syntax (Patel, 2003; Patel et al., 2008; Kunert et al., 2015). Electrophysiological studies found that patients with lesions in the left IFG show abnormal musical syntax processing and impaired behavioral performance in the processing of irregular chord sequences, which indicates the left IFG is the key region for the processing of syntax in a domain-general way (Patel et al., 2008; Sammler et al., 2011). Furthermore, music processing, like language processing, may involve shared dorsal and ventral neural networks, underlying structure and meaning processing, respectively, (Koelsch and Siebel, 2005; Musso et al., 2015). The dorsal stream – including IFG, anterior superior temporal gyrus (STG) and ventrolateral premotor cortex (PMC)—processes harmonic relations and structural irregularities, predicts short-term upcoming harmonic sequences (Koelsch and Siebel, 2005), and is involved independently in each type of musical stimuli (Tillmann et al., 2006). The left IFG further connects to the inferior parietal cortex and middle temporal lobe through dorsal and ventral long association tracts (Musso et al., 2015).

Although previous studies have provided a good basis for the understanding of music processing, so far most of the neuroscientific studies on music exclusively used Western classical music. Classical Western music is characterized by a strictly organized hierarchical structure, which may not be the case in other music genres. It is important, therefore, to examine a variety of music genres for providing a complete and unbiased picture of music processing (see also Brattico et al., 2013). Western art music has experienced a development stage from tonality to atonality. Tonal music is an interrelated sound system built around a tonal center or tonic, which is the foundation of music creation in the common composition period (i.e., from Bach to the end of the 19th century) and has formed a set of solid composition rules. Robert Francès (1988, pp. 122–127) provided evidence in support of “tonic traction” by transforming the degree of relevance between tonic and other notes (keys) into a spatial relationship. It is believed that all tonal music has a psychological pull toward the tonal center (Krumhansl, 1979).

A closer look at two other music genres, impressionist music and atonal music, shows those representative compositions of impressionism are partial to non-functional tonality or pandiatonicism. For example, in Debussy’s music, any notes in the tune that is simply emphasized and extended can be a new tonic. In this type of melody, the tonic is very flexible that can be endowed with a temporary tonic quality. Impressionist musicians such as Debussy divide an octave into six major second intervals of three kinds–major second, major third, and tritone (Day-O’Connell, 2009). Atonal music tends to specifically avoid pitch centrality and give up the tonal system of natural sounds. Schoenberg believed that it is necessary to prevent any set of tonic factors in any kind of harmonic relationship, regardless of whether it is vertical or horizontal and whether it is a phrase used in chord or chord progression. In “A Survivor of Warsaw” written by Schoenberg, the 12 semitones are functionally equal with no indication of the dominant tone of any sound, making it distinct from the major-minor system.

In short, the diatonic scale in impressionist music and the combination of 12 equal half-tones in atonal music both break the structural rules of classical music, either partially or completely. The asymmetry of the scale, the limitation of sound levels, and the size distribution of intervals within the scale are important factors that differentiate tonal, impressionist, and atonal music in music theory. According to the literature on music processing, the musical grammar would be disrupted if the interval relationship to the tonal center (i.e., pitch-center relationship) disappeared (Lerdahl and Jackendoff, 1983, chapters 3–4). The dissonance in music, which is unnatural to human ears, might cause inconsistent feelings or weariness for listeners (Lerdahl, 1988b, pp. 233–237). If this is the case for atonal music, we expect the neural networks underlying the processing of the regularities of pitch relationships and structure-based prediction to also work differently.

A further question is whether such neural activation is exclusively decided by the physical features of musical stimuli, which is identical for all listeners; or whether it rather reflects how the music is perceived by individuals, therefore, interacts with listeners’ music experience and preference. For example, for a non-trained listener, music may simply be a series of notes and beats, sometimes even a nuisance to the ear for lacking expectations (Daynes, 2010; Ockelford and Sergeant, 2012). For the romantic musician, in contrast, music can communicate just as well, or even better than language. In other words, music training and experience matter. Previous findings have shown that the early right anterior negativity (ERAN) ERP component is sensitive to music training (Koelsch et al., 2002b). A recent study further found that, in musicians, right IFG, right posterior STG, superior temporal sulcus (STS), and cerebellum were involved in the processing of the Western tonal musical structures, with resting state activity in right IFG positively correlated with that in posterior STG and left Heschl’s gyrus (Bianco et al., 2016). However, this study focused only on musicians, which in turn remains unclear how music experience modulates music processing and whether this process interacts with tonality.

The present study aimed to investigate the neural mechanisms underlying the processing of musical structure as well as the impact of tonality and expertise on such processing. To achieve this purpose, we included music genres that varied in tonality. Specifically, extending from previous studies focusing on music syntax processing of classical tonal music (Koelsch et al., 2013; Farbood et al., 2015), we also examined impressionist music (relatively decreased tonality) and atonal music (no tonality). A second aim of the present study was to investigate how musicianship modulated musical structure processing, and how it interacted with different music genres, that is, whether music experience affected brain networks underlying music tonal syntactic processing.

## 2. Methods

### 2.1. Participants

Thirty-six healthy native Chinese speakers with normal hearing, recruited from East China Normal University and Shanghai Conservatory of Music, took part in this study. All participants were right-handed, which is confirmed by using Edinburgh Handedness Inventory (Oldfield, 1971). Written informed consent was obtained from each participant, and the protocol of the present study was approved by the Committee on Human Research Protection at East China Normal University. All participants were paid for their participation.

The musicianship was determined using Music Experience Questionnaire. Half of the participants (*n* = 18) were musicians (22.4 ± 2.1 years, 16 females) who majored in instrumental (*n* = 17) or vocal performance (*n* = 1), and were immersed in classical music environments for on average 3.3 (± 2.4) hours per day. Five of the musicians reported having absolute pitch. They had on average 13 years (± 3.2 years, range 8–17 years) of formal music training, with an average age of onset of 5.5 years (± 1.3 years, range 3–8 years).

The other half of the participants (*n* = 18) were non-musicians (21.3 ± 3.3 years, 13 females) who reported no prior experience in music training, except one with 1-year experience in learning accordion and two with limited experience of playing piano or keyboard at young ages. The reason to include these three participants was based on their limited music experience and no music training in the last 10 years, but their data were excluded in further analysis.

In the questionnaire, the participants need to fill out how much they are familiar with the three music genres (rated from 1, not familiar to 5, very familiar). In the musician group, the average familiarity was scored 3.9 (SD = 0.5) for classic music, 2.2 (SD = 1.0) for impressionism music, and 1.8 (SD = 0.9) for atonal music. In the non-musician group, the average familiarity was scored 1.3 (SD = 0.6) for classic music. And for the other two music genres, the non-musicians all responded 1, not familiar. The t-test of musicianship and music familiarity was separately conducted in three music genres. The musicians were more familiar with all three music genres than non-musicians (classic music, *t*(34) = 13.8, *p* < 0.001; impressionism music, *t*(34) = 5.2, *p <* 0.001; atonal music, *t*(34) = 3.5, *p <* 0.01).

### 2.2. Materials

There were three experimental conditions for three genres of music (i.e., classical/tonal, impressionist/pantonal and atonal) and three control conditions. Scrambled versions corresponding for the three genres of music were used in the control conditions. To inspect more global and salient violations of tonal syntax, we adopted a method used in Levitin and Menon (2003), in which scrambled versions of musical pieces were included as baseline conditions to disrupt the musical structure.

Each of the three experimental conditions contained 40 phrases, selected from representative Western composers’ masterpieces, as listed in Table 1. The phrases were reconstructed using Sibelius software in order to be synchronous, to have a similar number of notes (32 ± 2 notes), and similar intensity. Only the relative positions of the notes or the internal organizational structure of the phrase were preserved. By doing so, the low-level acoustic features such as tempo, loudness, and timbre were balanced across music genres and left the music structure intact. The mean duration of the phrases was 6.2 (± 0.4) s. Scrambled versions were made by shuffling all of the notes within each of the original phrases so that the relative pitch of adjacent notes was disrupted. The scrambled phrases were then rated by three professional musicians independently to ensure that the inner original organizational structures had been destroyed while the same notes were kept. To increase the relative loudness of pitch in the noisy scanner environment, dynamic range compression was applied to all the pieces using the compressor effect of Audacity (Farbood et al., 2015).

**Table 1:** List of sources for the three genres of musical materials.

Genre	Composer	Catalogue	Number of phrases
Classical	Bach	The Well-Tempered Clavier	20
		BMV1043	
	Brahms	Hungarian Dances	20
		Symphony No.4	
Impressionist	Debussy	Estampes, Images, La Mer	20
		Prélude à l’après-midi d’un faune	
	Ravel	Miroirs, Gaspard de la nuit	20
		Ma mère l’Oye	
Atonal	Schoenberg	The Book of the Hanging Gardens	20
		String Quartets No. 3, Piano Suite	
	Webern	String Quartet, Variations	20

In addition to these stimuli, a 250-Hz pure tone (660 ms duration) was used as probe stimulus. Five such trials were included, inserted evenly between other trials, within each scanning session/run, to ensure that participants were attending to the task.

The music stimuli can be accessed through the project dataset on OSF platform^1^.

### 2.3. Procedure

**Figure 1:**
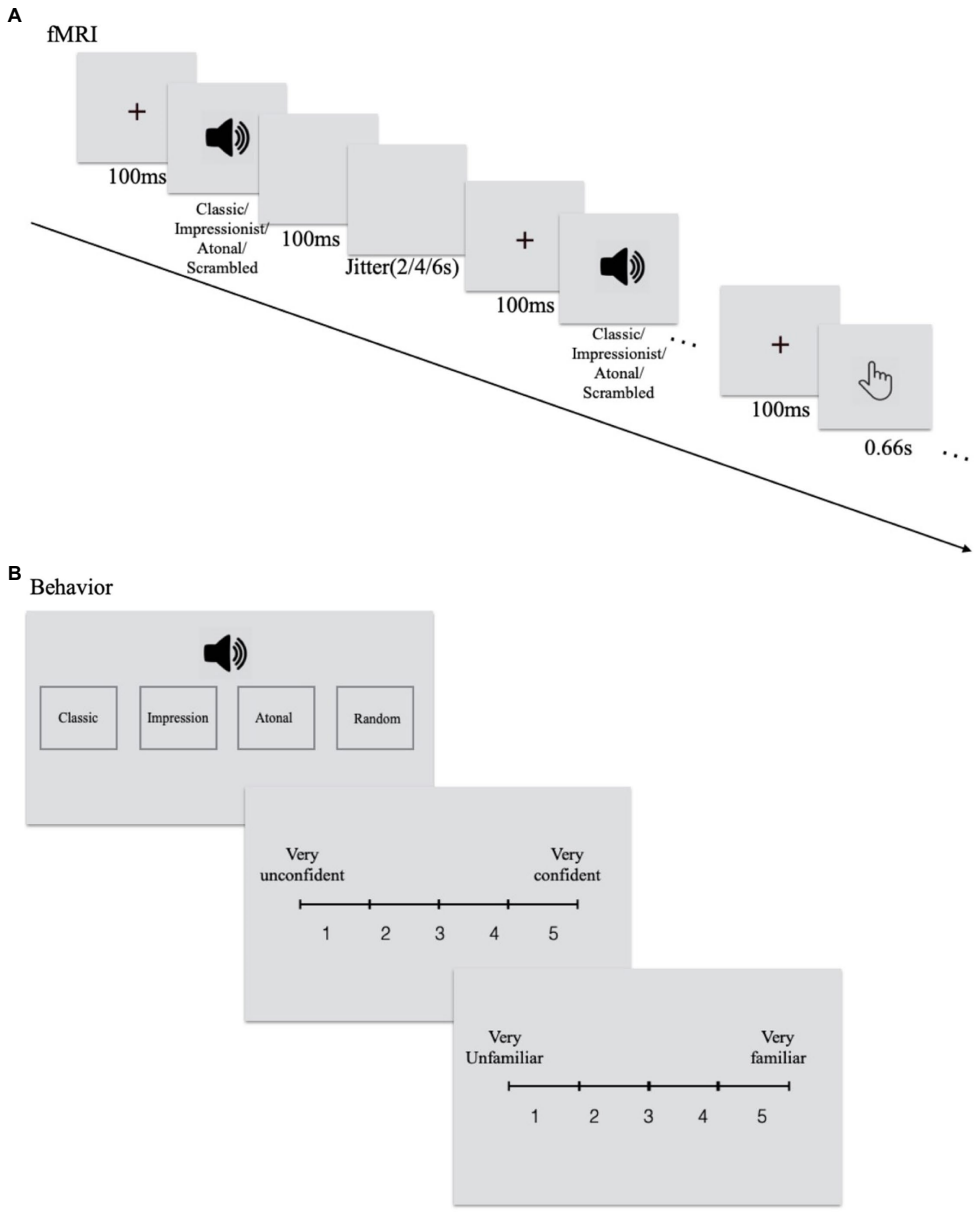
Experimental procedure for **(A)** the upper panel is fMRI and **(B)** the lower panel is behavioral tasks.

During fMRI scanning, participants were required to listen carefully to each phrase presented and they were not informed that some were original and others were scrambled. They were required to press the button with their right index finger when they heard the pure tone which had been presented to them outside before scanning. The same task was presented twice in the scanner. Each session/run contained 125 trials: 20 trials for each of the six conditions plus five pure-tone probe-detection trials. Each session/run started with a fixation of 10 s, and then all trials were presented in a random order. Between each phrase, a 2–4-6 s blank interval was presented (see Figure 1A). Stimuli were presented using E-Prime 2.0 software.

After scanning, participants listened to all phrases again, classified each piece into four categories (classical/tonal, impressionist, atonal music, and random notes), rated the level of confidence in his/their decision (from 1 = least confident to 5 = most confident), and familiarity with the phrase (from 1 = least familiar to 5 = most familiar; see Figure 1B).

### 2.4. Data acquisition

Whole-brain images were collected on a 3 T Siemens Trio MR scanner, with a 32-channel head coil. First, an anatomical image was obtained using a T1-weighted MPRAGE sequence (TR = 2,530 ms, TE = 2.34 ms, image matrix = 256 * 256, FoV = 256 mm, flip angle = 7°, voxel size = 1 * 1 * 1 mm, 192 slices). Functional MRI images were acquired using a T2*-weighted gradient-echo EPI sequence covering the whole brain (TR = 2,400 ms, TE = 30 ms, image matrix = 64 * 64, FoV = 192 mm, flip angle = 81°, voxel size = 3 * 3 * 3 mm, slice thickness = 3 mm, 40 slices, interleaved acquisition). Stabilization cushions were used to minimize head motion and ear plugs were worn by participants to reduce noise from the scanner during operation. Auditory stimuli were presented using RT-300 (Resonance Technology, Canada). Behavioral data were collected outside the MRI environment after scanning.

### 2.5. Behavioral data analysis

Two-way mixed design ANOVA with Tukey’s HSD comparison tests were performed separately for the genre classification, confidence rating, and familiarity rating, with group (musician, non-musician) and musical type (classical, impressionist, atonal, random notes) as independent factors. Data from two musicians were excluded because their accuracy for “random notes” were outliers (0 and 2.5%). Note that for each participant and each genre, the familiarity score was calculated based on ratings for all phrases, and the confidence score only took into account the correctly classified trials.

### 2.6. Functional imaging data analysis

Functional MRI data preprocessing and statistical analysis was carried out using SPM8.^2^ After slice-timing correction, the functional images were realigned for head motion correction. The functional and co-registered anatomical images were spatially normalized to MNI space, and then smoothed using a Gaussian kernel with full width at half maximum (FWHM) of 5 mm. Head movements were checked for each subject using Artifact Detection Tools (ART) package.^3^ Time points (scans/volumes) with motion outliers (≥ 2 mm) or outliers in global signal intensity (≥ 5 SD) were recorded for nine participants.

Data from each participant were then analyzed using a general linear model (GLM), with three musical genre conditions (classical, impressionist, and atonal), three scrambled conditions, and the probe condition. Head movement parameters were included for each participant as regressors, and the mentioned time points with motion or intensity outliers were omitted by including a single regressor for each in GLM. Familiarity scores from participants’ behavioral ratings were included as parametric modulators for each condition to dissociate familiarity effects from the main effects.

We first examined whether there were significant differences between any two scrambled conditions (out of the three scrambled conditions) using a 2 (groups) * 3 (genres) flexible factorial model at the group level. Given no significant main effect or interaction was found for the scrambled conditions, the three scrambled conditions were combined into one, referred to as the ‘random notes’ condition (matching the music genre classification in behavioral analysis). A 2 (groups) * 4 (musical structure: classical, impressionist, atonal, random notes) flexible factorial model was used in further analysis.

Given previous discoveries on the functional role of bilateral IFG in music processing, Anatomical ROIs of bilateral IFG (i.e., pars triangularis and pars opercularis) were selected from MarsBaR AAL ROIs. Percent signal change relative to global brain signal was computed using MarsBar, to further investigate how the brain reacted to different music genres in musicians and non-musicians.

Informational connectivity analysis (Coutanche and Thompson-Schill, 2013) was conducted to further derive the synchronous function of cortical regions that processed different music genres in musicians and non-musicians separately,. The whole brain was segmented into 116 regions of interest (ROIs) based on Automated Anatomical Labeling 116 (AAL116) template (Schmahmann et al., 1999; Tzourio-Mazoyer et al., 2002). Four ROIs were excluded in further analysis because they have not been fully covered in certain participants while scanning. For each ROI, a representational dissimilarity matrix (RDM) of all 240 musical trials was computed based on ß values extracted from all voxels for each participant. Then, for each ROI pair, the correlation coefficient was calculated between the two RDMs of the ROI pair and then transformed to fisher’s z values indicating representational similarity of general musical sentences processing between brain regions. After that, the correlation analysis was then performed separately for musicians and non-musicians to investigate the relationship between the *z* values of each region pair and the behavioral overall genre classification accuracy (representing each participant’s general musical genre sensitivity). Informational connectivity analysis allows us to inspect the highly stimuli-dependence neural processing between brain regions, which offers a higher-order explanation than univariate analysis.

## 3. Results

### 3.1. Behavioral results

**Figure 2:**
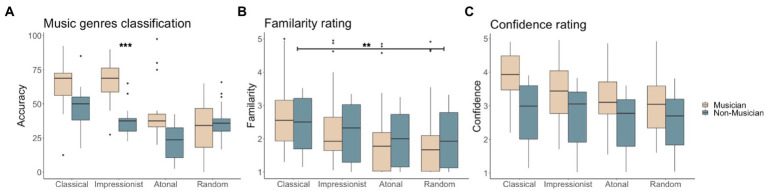
Behavioral results for musicians and non-musicians for **(A)** percentage correct genre classification, **(B)** familiarity ratings (1, least familiar ~ 5, most familiar) in musicians and non-musicians, and **(C)** confidence ratings (1, least confident ~ 5, most confident).

ANOVA on classification accuracy showed a main effect of group (*F*(1,136) = 25.058, *p* < 0.001), a main effect of music type (*F*(3,136) = 19.135, *p* < 0.001), and an interaction between group and musical type (*F*(3,136) = 4.837, *p* < 0.01). Tukey’s HSD post-hoc test indicated that classical music and impressionist music were easier to identify than atonal music (HSD = 24.118, *p* < 0.001, HSD = 20.881, *p* < 0.001, respectively classical and impressionist) and random notes (HSD = 15.662, *p* < 0.001, HSD = 12.425, *p* < 0.01, respectively for classical and impressionist). The musician group classified classical and impressionist music better than atonal music (HSD = 23.594, *p* < 0.001, HSD = 28.292, *p* < 0.001, respectively for classical and impressionist) and random notes (HSD = 22.552, *p* < 0.001, HSD = 27.25 *p* < 0.001, respectively for classical and impressionist). The non-musician group was found to have better knowledge only of classical compared to the atonal genre (HSD = 24.583, *p* < 0.001). Within music types, a significant group difference was only found for impressionist music classification, with musicians outperforming non-musicians (HSD = 28.083, *p* < 0.001; see Figure 2A).

For familiarity ratings, ANOVA showed only a significant main effect of music type (*F*(3,140) = 21.91, *p* < 0.001). *Post-hoc* tests showed that classical musical phrases were rated as significantly more familiar than atonal musical phrases (HSD = 0.56, *p* < 0.05), and significantly more familiar than random notes (HSD = 0.638, *p* < 0.01; see Figure 2B).

For confidence ratings, ANOVA showed significant main effects of group (*F*(3,142) = 9.079, *p* < 0.01) and of musical type (*F*(3,140) = 23.657, *p* < 0.001). Musicians were overall more confident than non-musicians in their genre classifications (HSD = 0.896, *p* < 0.001). Confidence was significantly higher when classifying classical music compared to atonal music (HSD = 0.727, *p* < 0.01) and random notes (HSD = 0.676, *p* < 0.01; see Figure 2C).

### 3.2. Functional imaging results

The group-level factorial analysis showed a significant interaction between group and musical structure, which involved activation in the right postcentral areas, left supplementary motor area (SMA), left middle temporal gyrus (MTG), left hippocampus, and bilateral superior frontal gyrus (SFG). The main effect of musical structure was observed in bilateral superior temporal regions, bilateral IFG pars triangularis extending to the left insula, bilateral superior medial frontal areas, bilateral precentral gyrus, right SFG, right middle frontal gyrus (MFG), left angular gyrus, right supramarginal gyrus, left SMA, and bilateral cerebellum. The main effect of group was observed in the bilateral cerebellum, bilateral precentral gyrus, right SFG, right superior temporal pole, bilateral inferior temporal gyrus, left amygdala, right STG, and bilateral IFG pars opercularis (all *p*’s < 0.001, alphasim corrected; see Table 2).

**Table 2:** Activation results of main effects of musical syntax and group, and simple effects of musicianship on classical and impressionist music processing (all alphasim corrected at *p* < 0.001).

Regions(aal)	ClusterSize	*Z*	*x* (mm)	*y* (mm)	*z* (mm)
*Musical syntax main effect*					
Temporal_Sup_L	282	7.04	−51	5	−5
Temporal_Mid_L		3.95	−54	−10	−17
Hippocampus_L	74	3.58	−27	−19	−20
Frontal_Sup_Medial_L	18	4.22	−6	59	13
Frontal_Inf_Tri_L	16	4.45	−36	23	−2
Insula_L	5	4.40	−39	17	4
Frontal_Mid_L	25	4.64	−24	23	37
Postcentral_L		3.60	−54	−13	37
Supp_Motor_Area_L	571	4.00	−6	2	64
Cerebelum_6_L		4.32	−30	−67	−23
Frontal_Mid_R	34	4.10	30	41	43
Frontal_Inf_Tri_R		4.58	51	32	19
Frontal_Sup_R	186	3.80	27	−7	61
Postcentral_R	377	4.05	54	−19	37
Pallidum_R	4	3.91	18	8	4
Cerebelum_Crus1_R	8	3.92	27	−85	−29
*Group main effect(musician > non-musician)*					
Cerebelum_Crus2_L	648	Inf	−12	−82	−32
Precentral_L	43	Inf	−21	−16	70
Parietal_Sup_L	37	6.41	−21	−67	40
Temporal_Mid_L	9	4.03	−63	−43	10
Temporal_Inf_L	34	7.51	−39	−43	−11
Frontal_Inf_Orb_L	5	4.74	−33	23	−11
Temporal_Inf_R	13	5.68	57	−46	−11
Frontal_Sup_R	6	7.20	15	47	22
Frontal_Inf_Orb_R	7	6.56	24	14	−11
Postcentral_R	8	6.20	48	−19	58
Cerebelum_6_R	12	5.80	24	−52	−26
Temporal_Pole_Sup_R	6	4.97	42	11	−20
*Classical syntax: musician > non-musician*					
Supp_Motor_Area_L	36	3.77	−6	17	46
Supp_Motor_Area_R		3.75	6	17	46
Frontal_Inf_Tri_R	8	3.41	45	20	4
Temporal_Sup_R	6	3.4	66	−22	4
Frontal_Sup_Medial_R		3.32	3	26	52
*Classical syntax: non-musician > musician*					
Cingulum_Ant_L	29	4.42	−3	32	1
Cingulum_Ant_R		3.71	0	26	−5
*Impressionist syntax: musician > non-musician*					
Vermis_9	13	4.52	0	−58	−32
*Impressionist syntax: non-musician > musician*					
Hippocampus_L	30	4.82	−30	−19	−20
Temporal_Mid_L	88	4.27	−51	−67	19
Frontal_Sup_L	17	3.57	−21	38	40
Postcentral_L	77	3.92	−57	−10	34
Frontal_Mid_R	29	3.88	27	29	34
Postcentral_R	76	4.33	54	−19	34

**Figure 3:**
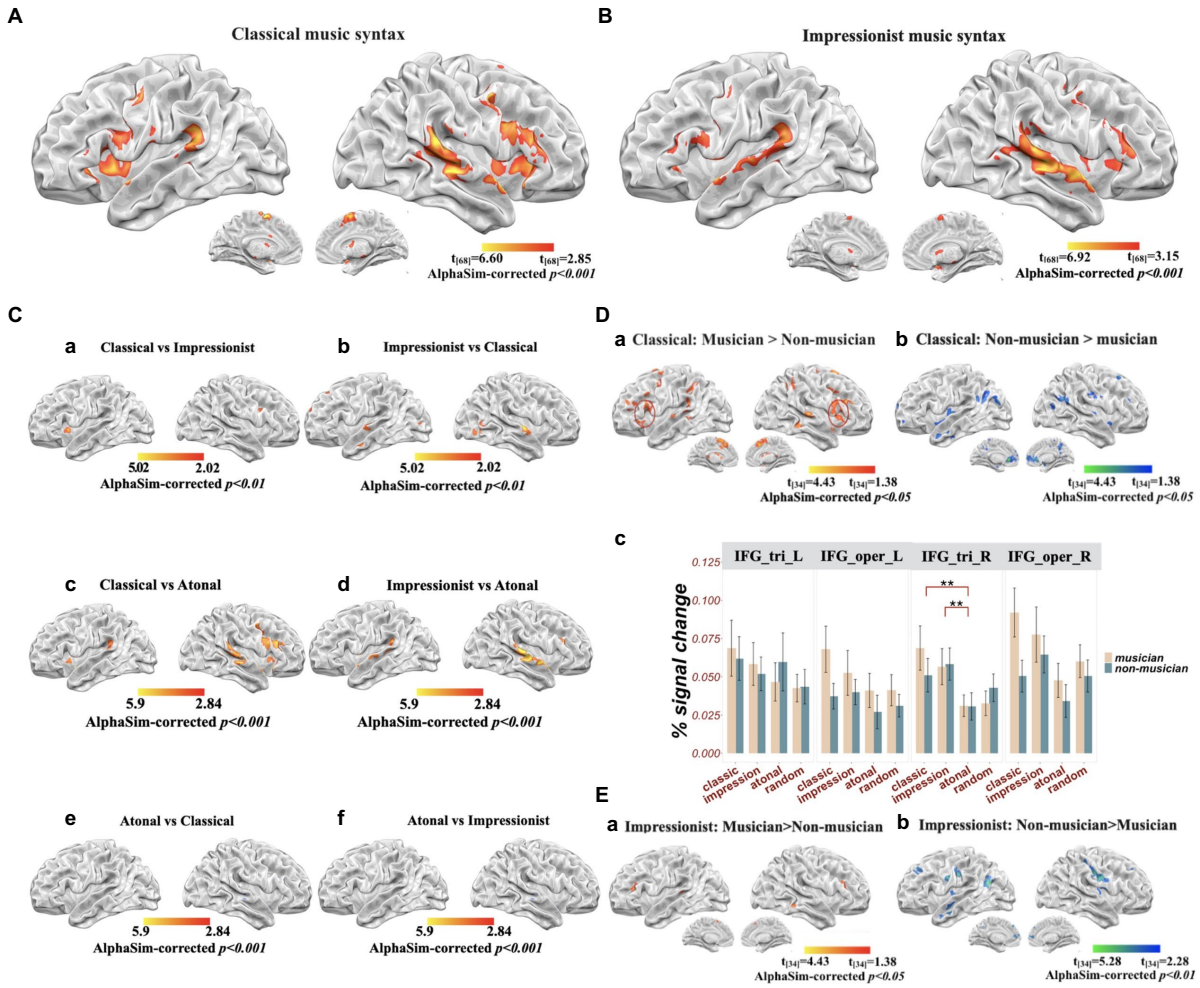
Brain activation for musical syntax processing (all results alphasim corrected at *p* < 0.001, unless otherwise stated). **(A)** Classical music compared to random notes; **(B)** impressionist music compared to random notes; **(C)** Comparisons among musical genres: (a) classical compared to impressionist music (alphasim corrected at *p* < 0.01 for illustration); (b) impressionist compared to classical music (alphasim corrected at *p* < 0.01 for illustration); (c) classical compared to atonal music; (d) impressionist compared to atonal music; (e) atonal compared to classical music; (f) atonal compared to impressionist music; **(D)** difference between groups for classical music: (a) musicians compared to non-musicians; (b) non-musicians compared to musicians; (c) percent signal change in left and right inferior frontal gyrus (IFG); **(E)** differences between groups for impressionist music: (a) musicians compared to non-musicians; (b) non-musicians compared to musicians.

Overall, classical/tonal music (compared to random notes) involved significant activation in the bilateral STG, left inferior frontal regions (including pars triangularis, pars opercularis, and pars orbitalis), right inferior frontal regions (including pars opercularis and insula), bilateral precentral gyrus, bilateral SMA, and bilateral cerebellum. Impressionist music (compared to random notes) involved significant activation in the bilateral STG, right superior temporal pole, right MTG, left IFG pars opercularis and pars triangularis, right IFG pars triangularis, left supramarginal gyrus, right hippocampus, right precentral gyrus, right SMA, and left cerebellum. When contrasting classical over impressionist music processing, the classical condition involved greater activation in the right IFG pars opercularis and left insula compared to the impressionist; the reverse contrast involved the right IFG pars triangularis, right precentral gyrus, bilateral STG, bilateral superior temporal pole, and left SMA. Compared to atonal music, classical music was more likely to be related to activation in the bilateral STG and MTG, right IFG pars triangularis and pars opercularis, left IFG pars opercularis and insula, bilateral precentral gyrus, bilateral SMA, and bilateral cerebellum; impressionist music showed more activation in the bilateral STG and MTG, left IFG pars triangularis and pars orbitalis, right IFG pars triangularis, bilateral SMA, bilateral putamen, and bilateral cerebellum. Atonal music involved more activation in bilateral MTG than classical music, without showing greater activation in any areas compared to impressionist music (all *p*’s < 0.001, AlphaSim corrected; see Figures 3A–C).

Simple effects were further analyzed using t-tests to investigate how the processing of musical structure was modulated by musicianship. For classical music processing, musicians showed greater activation in the right STG, right IFG pars triangularis, right superior medial frontal gyrus, right inferior parietal gyrus, and bilateral SMA, whereas bilateral anterior cingulate cortex (ACC) were more activated in non-musicians (all *p*’s < 0.001, AlphaSim corrected; see Table 2).

For processing impressionist music, musicians showed more activation in left cerebellum (Vermis 9) compared to non-musicians, yet non-musicians showed more activation in bilateral hippocampal gyrus, bilateral postcentral gyrus, MTG, SFG, insula, precuneus, and middle occipital lobe in the left hemisphere (all *p*’s < 0.001, AlphaSim corrected; see Table 2, Figure 3E).

Lastly, for atonal music, no significant differences were found between musicians and non-musicians (*p*’s < 0.001, AlphaSim corrected).

For the ROI analysis on the bilateral IFG (see Figure 3D(c)), left pars triangularis showed no significant effects of music genre (*F*_(3)_ = 0.842, *p* = 0.472) or group (*F*_(1)_ = 0.000, *p* = 0.984), or their interaction (*F*_(1,3)_ = 0.218, *p* = 0.884). A significant musical structure main effect (*F*_(1,3)_ = 0.048, *p* < 0.01) was found for right pars triangularis, specifically, both classical (*t* = 2.76, *p* < 0.01, 95% CI = [0.0112,0.0222]) and impressionist music (*t* = 3.28, *p* < 0.01, 95% CI = [0.0683,0.0745]) had greater signal change than atonal music. For both left and right pars opercularis, there were significant group differences (left: *F*_(1,3)_ = 4.61, *p* < 0.05; right: *F*_(1,3)_ = 4.65, *p* < 0.05), with percent signal change in musicians greater than in non-musicians (left: *t* = 2.15, *p* < 0.05, 95% CI = [0.0014,0.0322]; right: *t* = 2.18, *p* < 0.05, 95% CI = [0.0553,0.0459]).

**Figure 4:**
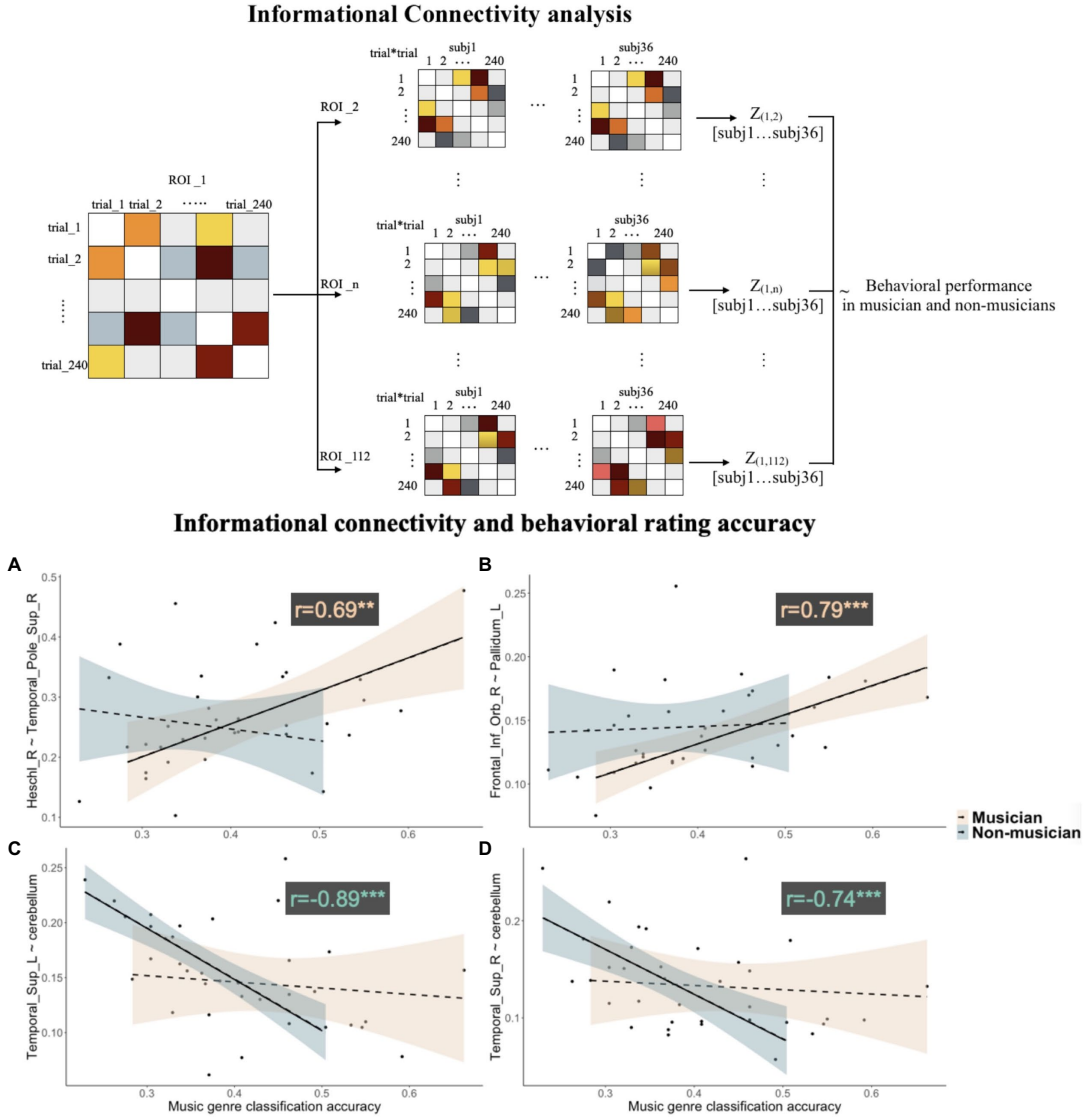
Upper panel: illustration for computing informational connectivities between ROIs for all the participants. Lower panel: correlations between informational connectivities and behavioral classification accuracy in musicians and non-musicians, for **(A)** connectivity between right Heschl’s gyrus and right superior temporal pole, **(B)** connectivity between right IFG pars orbitalis and left pallidum, **(C)** connectivity between the left superior temporal gyrus and cerebellum, and **(D)** connectivity between right superior temporal gyrus and cerebellum.

Informational connectivity between the right Heschl’s gyrus and right superior temporal pole was positively correlated with behavioral classification accuracy in musicians (*r* = 0.69, FDR corrected at *q* = 0.005); informational connectivity between the right IFG pars orbitalis and left pallidum was also positively correlated with behavioral classification accuracy in musicians (*r* = 0.79, FDR corrected at *q* = 0.005). Informational connectivity between the cerebellum (cerebellar vermis 7, VER7) and both left and right STG was negatively correlated with behavioral accuracy in non-musicians (left STG: *r* = −0.89, *q* = 0.0001; right STG: *r* = −0.74, *q* = 0.0001; see Figure 4).

## 4. Discussion

The present study investigated the neural mechanisms underlying tonality and musical syntax/structure processing, as well as the role of music training in such processing. Musicians and non-musicians listened to phrases from classical, impressionist, and atonal music genres inside an MRI scanner, and performed a classification task outside the scanner. The results elucidated the online processing mechanisms of musical syntax across different genres and demonstrated how musicianship impacted the neural responses to different musical syntax.

### 4.1. Musical structure, tonality, and musicianship

For the overall processing of hierarchical structure in music, the neural response was observed in the bilateral temporal lobes, IFG, postcentral gyrus, and cerebellum. This finding indicates the engagement of the dorsal stream in decoding musical structure where the auditory information is transformed into motor actions. This engagement may be stronger among musicians than non-musicians in the presence of tonality, as discussed later.

For Western classical music perception, musicians and non-musicians both achieved high accuracy in behavioral classification while musicians tended to have higher confidence ratings, suggesting that musicians took advantage of their expertise to analyze the musical notes. The bilateral anterior superior temporal areas, bilateral left inferior frontal regions extending to bilateral precentral gyrus, insula, SMA, and cerebellum were engaged in the processing of tonal music, which is in line with previous studies (Koelsch et al., 2002a, 2013; Tillmann et al., 2006; Sammler et al., 2013; Farbood et al., 2015). Previous studies suggested that people tended to perceive musical syntax implicitly regardless of music training (Koelsch et al., 2000; Bigand and Poulin-Charronnat, 2006). In contrast to these studies, our results found that music experience modulated neural activation in classical tonal music processing while non-musicians and musicians performed equally well in behavioral classifications. Specifically, differences between musicians and non-musicians in neural activation were observed in a right-lateralized front-parieto-tempral network covering the right STG, right IFG pars triangularis and superior medial frontal gyrus, right inferior parietal gyrus, and bilateral SMA. Together with previous studies showing the role of the right IFG in musical syntax processing (Cheung et al., 2018) and structural brain changes in the right fronto-temporal regions linked to music training (James et al., 2014; Sato et al., 2015), the present findings suggest that the left fronto-temporal neural network may play an important role in musical syntactic processing in a domain-general and experience-independent way, and that the right fronto-temporal cortical areas may contribute to musical syntactic processing in a musicianship-modulated way.

For impressionist music, musicians showed significantly higher accuracy in behavioral classification, as well as stronger activation in the left cerebellum than non-musicians. A closer look at the neural basis among musicians and non-musicians revealed that the bilateral STG and bilateral IFG pars triangularis were engaged in both groups, whereas the right IFG was significantly recruited only among musicians. These results suggest that the minor disruption of tonality rules in impressionist music could weaken the functions of the left IFG in resolving musical syntax. The right IFG, on the other hand, still tends to play an important role in musical syntax processing, particularly with music training. Together with the results of classical music processing, these results indicate that music experience could have an impact on the neural response to syntactic processing of tonal music—both classical tonal and impressionist (reduced tonality).

For atonal music, there were no differences between musicians and non-musicians in either neural activation or behavioral classification performance. Furthermore, atonal music could not be differentiated from random notes (either neurally or behaviorally) even among musicians. This is likely due to a lack of pitch-center relationship in atonal music, leading to an absence of structural information processing. Given that previous studies on atonal music suggested that familiarity has some effects on induced emotional responses to atonal music (Daynes, 2000), or that listeners can learn to detect or expect the avoidance of pitch repetition (Krumhansl et al., 1987; Ockelford and Sergeant, 2012), it would be of interest for future studies to investigate whether atonal music could be processed differently among musicians with more varied experiences and those with expertise in atonal music, such as music composers and conductors who have developed a positive taste for atonal music.

### 4.2. Cortical and subcortical neural networks for musical structure processing

The IFG has been deemed to be a storage buffer required to process sequences with supra-regular structure (Fitch and Martins, 2014). Within the IFG, left pars triangularis, a part of Broca’s area, has been suggested to be involved in domain-general processing, playing a crucial role in sequence regularities, and particularly being the site of a buffer zone for syntactic computations (Sammler et al., 2011; Fitch and Martins, 2014). Previous studies have further put forward a shared resource system for domains of both language and music, seated in Broca’s area (Patel, 2003; Fedorenko et al., 2009). The role of the right IFG is less clear, though some studies have suggested that the right inferior frontal area is crucial for processing specific musical syntax (Maess et al., 2001), and is sensitive to music training (Koelsch et al., 2002b; Oechslin et al., 2013). In the present study, the left pars triangularis was engaged in the syntactic processing of classical music equally for musicians and non-musicians. The right pars triangularis and pars opercularis, on the other hand, were involved to a greater extent among musicians compared to non-musicians in the syntactic processing of both classical and impressionist music. Percent signal change of different subregions of the bilateral IFG further showed that right pars triangularis was sensitive to tonal differences, and both left and right pars opercularis were sensitive to music experience differences. We therefore suggest a more precise division of labor of the bilateral IFG regions in music processing: the left IFG pars triangularis carries out on-line unit relationship computations independently of music genre and music experience; the right IFG pars triangularis detects tonality and adjusts to tonal varieties, partly relying on music experience; both left and right pars opercularis (more dominantly) are modulated by music experience.

We also found involvement of the right anterior temporal regions and right frontal regions in musical syntactic processing, especially among musicians. Furthermore, informational connectivity results revealed that higher behavioral classification accuracy among musicians was accompanied by stronger functional cooperation between the right Heschl’s gyrus and right superior temporal pole. According to previous findings, temporal resolution is in favor of left auditory cortices, whereas spectral resolution is better in right auditory cortices (Zatorre et al., 2002). Therefore, our results suggest that right temporal regions are more likely to be engaged in musicians to achieve better performance in detecting precise changes in frequency. Together with abovementioned results on frontal regions, the present findings suggest that a right fronto-temporal network is crucial in allowing musicians to outperform non-musicians in musical syntactic processing.

The neural processing of musical structure engages not only cortical structures but also subcortical structures, such as basal ganglia, which has been found to be activated in the processing of musical beats and music-related emotions (Frisch et al., 2003; Kung et al., 2013). In the present study, neural recruitment of pallidum and the cerebellum was found for processing tonal music in musicians. Results of the informational connectivity analysis showed that strong connectivity between the right IFG and left pallidum was positively correlated with music classification performance in musicians. Given that the sensorimotor territory of the globus pallidus internus is known to be the main output of basal ganglia, the region for the storage and expression of learned sequential skills (Hikosaka et al., 2002; Doyon et al., 2009), the current finding of pallidum activation and its connection with the right IFG is especially interesting. Furthermore, both globus pallidus and cerebellum appear to be the most effective sites for deep brain simulation (DBS) in reducing motor impairments (Tewari et al., 2017). A recent study also found that the basal ganglia and the cerebellum were interconnected at the subcortical level (Bostan and Strick, 2018). Therefore, our findings suggest that this cortico-subcortical network may facilitate the perception of musical sequences, especially for the musicians due to their intensive training in music performance.

The left cerebellum was also found to be significantly more engaged in musicians compared to non-musicians in the processing of impressionist music. Among the non-musicians, connectivity between the cerebellum and bilateral STG was negatively correlated with classification performance. A previous study suggested that experience-dependent changes in the cerebellum could contribute to motor sequence learning (Doyon et al., 2002). Given that the motor network is important for production and perception of music (Schubotz et al., 2000), our results for the musicians suggest that the engagement of cerebellum may facilitate motor sequence and musical sequence perception in turn. Further studies are needed to clarify the role of the cerebellum-STG connectivity in music processing among non-musicians.

A cortico-subcortical network involving the putamen, SMA, and PMC has been proposed to be engaged in the analysis of temporal sequences and in auditory–motor interactions (Grahn and Rowe, 2009). The present study provided further evidence on the engagement of these proposed regions. In addition, the current findings allowed us to have a more refined understanding of the functions of different regions. Furthermore, this cortical–subcortical connectivity is shown to be functionally correlated with behavioral performance in music genre classification and neural musical syntax processing among musicians.

### 4.3. Appreciation of tonality in music from a scientific perspective

Western classical (tonal) music has been widely appreciated due to its consonance and stability. In the present study, musicians showed stronger and more widespread neural responses to classical music compared to non-musicians. Non-musicians, though with relatively less activation than musicians, still showed stronger neural responses to classical music than to impressionist or atonal music. The higher accuracy in classifying classical musical phrases among non-musicians can be seen as evidence of implicit knowledge of musical structure even among those with minimal musical expertise. Furthermore, as described by Tonal Pitch Space (TPS) theory (Lerdahl, 1988a), the tension and relaxation of chords unfolding over time in classical music provide listeners with a musical context in which to generate reliable expectations.

Impressionist music, on the other hand, is well-known for feelings of ambiguity and intangibility, like impressionist paintings. This music genre places the listener in a reduced tonality context, which leads to difficulty in integrating harmonics. Although impressionist music and classical music both engaged similar frontotemporal regions, they tended to related to different specific regions as well suggested by the current study. Furthermore, the differences between musicians and non-musicians in both behavioral and neural responses suggest that the processing of impressionist music may especially involve the frontal regions of the right hemisphere, and impressionist music processing could benefit from musicianship more so than classical music processing.

Lastly, the atonal genre stands opposite to tonality. Its disordered structure and unexpected musical context may well be perceived as scrambled pieces, resulting in poor performance in differentiating atonal phrases from random notes, and in a lack of significant differences in neural responses between atonal phrases and random notes, regardless of the level of music experience. There are only a few studies on tonality in neuroscience. Among them, Proverbio et al. (2015) suggested that atonal music decreased non-musicians heart rates and increased their blood pressure, possibly reflecting an increase in alertness and attention, and thus appeared to be perceived as being more agitating and less joyful than tonal music. The present study provides complementary results regarding the absence of “syntactic” processing in atonal music perception and questions the “meaning” of atonal music.

However, there are two limitations of the current study. First, controlling the music from different periods and the composers’ personal characteristics may induce concerns about generalizing to other music works. For example, it is debatable to treat Brahms and Bach’s music as representatives of Western classic tonal music since they came from different periods. However, fundamentally, Brahms, a solid defender of classical music, has an internal consistency with Bach’s music from the perspective of the functional tonality. Besides, such within-condition differences could indeed better support the results of any existing between-group differences. A second limitation of the scrambled paradigm in the current study is the melodic contours, which may have an interactive connection with music syntax. While in the current study, we emphasized the psychological completeness following by listening to music with structures. Further investigations should put emphasis on the differences in melodic contours in recognition of various musical genres.

Overall, this study made efforts to explore the musical structure processing in different music genres with varying tonality behaviorally and neurally, and the modulation effect of musical training experience. Previous findings on neural mechanism of music syntax mainly came from the inspection of the tonality structure in Western classical tonal music. The current project incorporating music theory tried to elicit more discussion on the tonality varieties in other music genres and generalize the neural mechanism of music syntax to a broader musical context. While many previous studies on musical syntax have focused on the left prefrontal cortex and compared it to the neural basis of syntactic processing in language, the results of the current study demonstrate that the right prefrontal cortex may play more important roles in a complex interaction between tonality varieties and music experience.

## Data availability statement

The datasets presented in this study can be found in online repositories. The names of the repository/repositories and accession number(s) can be found at: https://osf.io/4fejw/.

## Ethics statement

The studies involving human participants were reviewed and approved by Committee on Human Research Protection at East China Normal University. The patients/participants provided their written informed consent to participate in this study.

## Author contributions

LJ, YoZ, and QC conceived the original idea and verified the findings and provided the experimental facilities. LJ, RZ, and QC designed the study, and wrote the original manuscript. LJ and RZ collected the data and performed the analyzes. RZ, LT, YuZ, YoZ, and QC discussed the results and revised the manuscript. All authors contributed to the article and approved the submitted version.

## Funding

This work was supported by Art Project of National Social Science Foundation of China (No. 16BD050), National Natural Science Foundation of China (Nos. 31970987 and 31771210), National Social Science Major Project of China (No. 17ZDA323), and Science and Technology Commission of Shanghai Municipality (No. 19JC1410101). We also appreciate Shuai Wang’s suggestions for informational connectivity analysis.

## Conflict of interest

The authors declare that the research was conducted in the absence of any commercial or financial relationships that could be construed as a potential conflict of interest.

## Publisher’s note

All claims expressed in this article are solely those of the authors and do not necessarily represent those of their affiliated organizations, or those of the publisher, the editors and the reviewers. Any product that may be evaluated in this article, or claim that may be made by its manufacturer, is not guaranteed or endorsed by the publisher.

## Footnotes

^1^https://osf.io/4fejw/

^2^www.fil.ion.ucl.ac.uk/spm

^3^www.nitrc.org/projects/artifact_detect

## References

[ref1] Bianco R. Novembre G. Keller P. E. Kim S.-G. Scharf F. Friederici A. D.. (2016). Neural networks for harmonic structure in music perception and action. NeuroImage. 142, 454–464. doi: 10.1016/j.neuroimage.2016.08.025, PMID: 27542722

[ref2] Bigand E. Poulin-Charronnat B. (2006). Are we “experienced listeners”? A review of the musical capacities that do not depend on formal musical training. Cognition 100, 100–130. doi: 10.1016/j.cognition.2005.11.007, PMID: 16412412

[ref3] Bostan A. C. Strick P. L. (2018). The basal ganglia and the cerebellum: nodes in an integrated network. Nat. Rev. Neurosci. 19, 338–350. doi: 10.1038/s41583-018-0002-7, PMID: 29643480 PMC6503669

[ref4] Brattico E. Bogert B. Jacobsen T. (2013). Toward a neural chronometry for the aesthetic experience of music. Front. Psychol. 4:206. doi: 10.3389/fpsyg.2013.00206 23641223 PMC3640187

[ref5] Cheung V. K. M. Meyer L. Friederici A. D. Koelsch S. (2018). The right inferior frontal gyrus processes nested non-local dependencies in music. Sci. Rep. 8, 1–12. doi: 10.1038/s41598-018-22144-9 29491454 PMC5830458

[ref6] Coutanche M. N. Thompson-Schill S. L. (2013). Informational connectivity: identifying synchronized discriminability of multi-voxel patterns across the brain. Front. Hum. Neurosci. 7:15. doi: 10.3389/fnhum.2013.00015 23403700 PMC3566529

[ref7] Daynes H. (2010). Listeners’ perceptual and emotional responses to tonal and atonal music. Psychol. Music 39, 468–502. doi: 10.1177/0305735610378182

[ref8] Day-O’Connell J. (2009). Debussy, Pentatonicism, and the tonal tradition. Music Theory Spectrum. 31, 225–261. doi: 10.1525/mts.2009.31.2.225

[ref9] Doyon J. Bellec P. Amsel R. Penhune V. Monchi O. Carrier J.. (2009). Contributions of the basal ganglia and functionally related structures to motor learning. Behav. Brain Res. 199, 61–75. doi: 10.1016/j.bbr.2008.11.012 19061920

[ref10] Doyon J. Song A. W. Karni A. Lalonde F. Adams M. M. Ungerleider L. G. (2002). Experience-dependent changes in cerebellar contributions to motor sequence learning. Proc. Natl. Acad. Sci. 99, 1017–1022. doi: 10.1073/pnas.022615199, PMID: 11805340 PMC117423

[ref11] Farbood M. M. Heeger D. J. Marcus G. Hasson U. Lerner Y. (2015). The neural processing of hierarchical structure in music and speech at different timescales. Front. Neurosci. 9:157. doi: 10.3389/fnins.2015.00157 26029037 PMC4429236

[ref12] Fedorenko E. Patel A. Casasanto D. Winawer J. Gibson E. (2009). Structural integration in language and music: evidence for a shared system. Mem. Cogn. 37, 1–9. doi: 10.3758/MC.37.1.1, PMID: 19103970

[ref13] Feng L. Wang X. (2017). Harmonic template neurons in primate auditory cortex underlying complex sound processing. Proc. Natl. Acad. Sci. 114, E840–E848. doi: 10.1073/pnas.1607519114, PMID: 28096341 PMC5293092

[ref14] Fitch W. T. Martins M. D. (2014). Hierarchical processing in music, language, and action: Lashley revisited. Ann. N. Y. Acad. Sci. 1316, 87–104. doi: 10.1111/nyas.12406, PMID: 24697242 PMC4285949

[ref15] Francès R. (1988). The Perception of Music (Trans. J. Dowling). Hillsdale, NJ. Lawrence Erlbaum.

[ref16] Frisch S. Kotz S. A. von Cramon D. Y. Friederici A. D. (2003). Why the P600 is not just a P300: the role of the basal ganglia. Clin. Neurophysiol. 114, 336–340. doi: 10.1016/S1388-2457(02)00366-8 12559242

[ref17] Grahn J. A. Rowe J. B. (2009). Feeling the beat: premotor and striatal interactions in musicians and nonmusicians during beat perception. J. Neurosci. 29, 7540–7548. doi: 10.1523/JNEUROSCI.2018-08.2009, PMID: 19515922 PMC2702750

[ref18] Hikosaka O. Nakamura K. Sakai K. Nakahara H. (2002). Central mechanisms of motor skill learning. Curr. Opin. Neurobiol. 12, 217–222. doi: 10.1016/S0959-4388(02)00307-0 12015240

[ref19] James C. E. Oechslin M. S. Van De Ville D. Hauert C. A. Descloux C. Lazeyras F. (2014). Musical training intensity yields opposite effects on grey matter density in cognitive versus sensorimotor networks. Brain Struct. Funct. 219, 353–366. doi: 10.1007/s00429-013-0504-z, PMID: 23408267

[ref20] Koelsch S. Gunter T. Friederici A. D. Schröger E. (2000). Brain indices of music processing: “nonmusicians” are musical. J. Cogn. Neurosci. 12, 520–541. doi: 10.1162/089892900562183 10931776

[ref21] Koelsch S. Gunter T. C. v Cramon D. Y. Zysset S. Lohmann G. Friederici A. D. (2002a). Bach speaks: a cortical “language-network” serves the processing of music. NeuroImage. 17, 956–966. doi: 10.1006/nimg.2002.1154 12377169

[ref22] Koelsch S. Rohrmeier M. Torrecuso R. Jentschke S. (2013). Processing of hierarchical syntactic structure in music. Proc. Natl. Acad. Sci. 110, 15443–15448. doi: 10.1073/pnas.1300272110, PMID: 24003165 PMC3780886

[ref23] Koelsch S. Schmidt B. Kansok J. (2002b). Effects of musical expertise on the early right anterior negativity: an event-related brain potential study. Psychophysiology 39, 657–663. doi: 10.1111/1469-8986.3950657, PMID: 12236333

[ref24] Koelsch S. Siebel W. A. (2005). Towards a neural basis of music perception. Trends Cogn. Sci. 9, 578–584. doi: 10.1016/j.tics.2005.10.001, PMID: 16271503

[ref25] Krumhansl C. L. (1979). The psychological representation of musical pitch in a tonal context. Cogn. Psychol. 11, 346–374. doi: 10.1016/0010-0285(79)90016-1

[ref26] Krumhansl C. L. Sandell G. J. Sergeant D. C. (1987). The perception of tone hierarchies and mirror forms in twelve-tone serial music. Music. Percept. 5, 31–77. doi: 10.2307/40285385

[ref27] Kunert R. Willems R. M. Casasanto D. Patel A. D. Hagoort P. (2015). Music and language syntax interact in Broca’s area: an fMRI study. PLoS One 10:e0141069. doi: 10.1371/journal.pone.0141069, PMID: 26536026 PMC4633113

[ref28] Kung S.-J. Chen J. L. Zatorre R. J. Penhune V. B. (2013). Interacting cortical and basal ganglia networks underlying finding and tapping to the musical beat. J. Cogn. Neurosci. 25, 401–420. doi: 10.1162/jocn_a_00325, PMID: 23163420

[ref29] Lerdahl F. (1988a). Tonal pitch space. Music Perception 5, 315–349. doi: 10.2307/40285402

[ref30] Lerdahl F. (1988b). “Cognitive constraints on compositional systems,” in Generative Processes in Music: The Psychology of Performance, Improvisation and Composition. ed. Sloboda J. (Oxford: Clarendon Press), 231–259. doi: 10.1093/acprof:oso/9780198508465.001.0001

[ref31] Lerdahl F. Jackendoff R. (1983). A Generative Theory of Tonal Music. Cambridge, MA: MIT Press.

[ref32] Levitin D. J. Menon V. (2003). Musical structure is processed in “language” areas of the brain: a possible role for Brodmann area 47 in temporal coherence. NeuroImage. 20, 2142–2152. doi: 10.1016/j.neuroimage.2003.08.016, PMID: 14683718

[ref33] Maess B. Koelsch S. Gunter T. C. Friederici A. D. (2001). Musical syntax is processed in Broca’s area: an MEG study. Nat. Neurosci. 4, 540–545. doi: 10.1038/87502, PMID: 11319564

[ref34] Musso M. Weiller C. Horn A. Glauche V. Umarova R. Hennig J.. (2015). A single dual-stream framework for syntactic computations in music and language. NeuroImage. 117, 267–283. doi: 10.1016/j.neuroimage.2015.05.020, PMID: 25998957

[ref35] Ockelford A. Sergeant D. (2012). Musical expectancy in atonal contexts: Musicians' perception of "antistructure". Psychol. Music 41, 139–174. doi: 10.1177/0305735612442582

[ref36] Oechslin M. S. Van De Ville D. Lazeyras F. Hauert C.-A. James C. E. (2013). Degree of musical expertise modulates higher order brain functioning. Cereb. Cortex 23, 2213–2224. doi: 10.1093/cercor/bhs206, PMID: 22832388

[ref1001] Oldfield R. C. (1971). The assessment and analysis of handedness: the Edinburgh inventory. Neuropsychologia 9, 97–113. doi: 10.1016/0028-3932(71)90067-4 5146491

[ref37] Patel A. D. (2003). Language, music, syntax and the brain. Nat. Neurosci. 6, 674–681. doi: 10.1038/nn1082 12830158

[ref38] Patel A. D. Iversen J. R. Wassenaar M. Hagoort P. (2008). Musical syntactic processing in agrammatic Broca's aphasia. Aphasiology 22, 776–789. doi: 10.1080/02687030701803804

[ref39] Proverbio A. M. Manfrin L. Arcari L. A. De Benedetto F. Gazzola M. Guardamagna M.. (2015). Non-expert listeners show decreased heart rate and increased blood pressure (fear bradycardia) in response to atonal music. Front. Psychol. 6:1646. doi: 10.3389/fpsyg.2015.01646, PMID: 26579029 PMC4623197

[ref40] Sammler D. Koelsch S. Friederici A. D. (2011). Are left fronto-temporal brain areas a prerequisite for normal music-syntactic processing? Cortex 47, 659–673. doi: 10.1016/j.cortex.2010.04.007, PMID: 20570253

[ref41] Sammler D. Novembre G. Koelsch S. Keller P. E. (2013). Syntax in a pianist’s hand: ERP signatures of “embodied” syntax processing in music. Cortex 49, 1325–1339. doi: 10.1016/j.cortex.2012.06.007, PMID: 22832238

[ref42] Sato K. Kirino E. Tanaka S. (2015). A voxel-based Morphometry study of the brain of university students majoring in music and nonmusic disciplines. Behav. Neurol. 2015, 1–9. doi: 10.1155/2015/274919 PMC4606127 26494943

[ref43] Schmahmann J. D. Doyon J. McDonald D. Holmes C. Lavoie K. Hurwitz A. S.. (1999). Three-dimensional MRI atlas of the human cerebellum in proportional stereotaxic space. NeuroImage. 10, 233–260. doi: 10.1006/nimg.1999.0459 10458940

[ref44] Schubotz R. I. Friederici A. D. von Cramon D. Y. (2000). Time perception and motor timing: a common cortical and subcortical basis revealed by event-related fMRI. NeuroImage. 11, 1–12. doi: 10.1006/nimg.1999.0514, PMID: 10686112

[ref45] Tewari A. Fremont R. Khodakhah K. (2017). It’s not just the basal ganglia: cerebellum as a target for dystonia therapeutics. Mov. Disord. 32, 1537–1545. doi: 10.1002/mds.27123, PMID: 28843013 PMC5815386

[ref46] Tillmann B. Koelsch S. Escoffier N. Bigand E. Lalitte P. Friederici A. D.. (2006). Cognitive priming in sung and instrumental music: activation of inferior frontal cortex. NeuroImage. 31, 1771–1782. doi: 10.1016/j.neuroimage.2006.02.028, PMID: 16624581

[ref47] Tzourio-Mazoyer N. Landeau B. Papathanassiou D. Crivello F. Etard O. Delcroix N.. (2002). Automated anatomical labeling of activations in SPM using a macroscopic anatomical parcellation of the MNI MRI single-subject brain. NeuroImage. 15, 273–289. doi: 10.1006/nimg.2001.0978, PMID: 11771995

[ref48] Zatorre R. J. Belin P. Penhune V. B. (2002). Structure and function of auditory cortex: music and speech. Trends Cogn. Sci. 6, 37–46. doi: 10.1016/S1364-6613(00)01816-7 11849614

